# Noisy Memory Generates Value in Changing Environments

**DOI:** 10.1177/10597123251372839

**Published:** 2025-09-08

**Authors:** Jorge Ramírez-Ruiz, R. Becket Ebitz

**Affiliations:** 1 Département de Neurosciences, Faculté de Médecine, 5622 Université de Montréal, Montréal, QC, Canada

**Keywords:** memory errors, probabilistic choice, value integration

## Abstract

Experimental data suggest that episodic memory is involved in sequential value-based decision-making. By contrast, standard computational models of decision-making assume that prior reward outcomes are integrated into subjective values rather than remembered discretely. Previous work developed a minimal computational framework for sequential value-based decision-making that is based on noisy sampling of episodic memories, rather than calculating value. We called these agents “Imperfect Memory Programs” (IMPs) and showed how their single free parameter optimizes the trade-off between the magnitude of error and the complexity of imperfect recall. Here, we develop biologically plausible approximations to the IMPs with lossy agents (LIMPs) that maintain only 1 bit of reward memory for binary outcomes but fail to encode rewards with some probability. Both IMPs and LIMPs perform similarly to or better than a simple agent with perfect memory in multiple classic decision-making tasks and generate phenomenology that resembles value-based computations. We find that allowing different encoding probabilities for rewards and omissions improves performance further and allows to trade-off matching versus maximizing behavior, as well as flexible versus stable performance. Together, these results suggest that episodic agents can approximate value-based agents through capitalizing on realistic encoding and/or sampling noise. This suggests that mnemonic errors (1) can improve, rather than impair decision-making and (2) provide a plausible alternative explanation for some behavioral correlates of “value”.

## Introduction

There is growing evidence in the cognitive and neural sciences that episodic memory plays an important role in sequential value-based decisions. Not only can people recognize discrete instances of individual prior reward outcomes out of streams of hundreds of such outcomes (Bornstein et al., 2017; Cohen et al., 2022; Nicholas et al., 2022), but people also seem to use remembered episodes to guide decisions (Bornstein et al., 2017; Cohen et al., 2022). While there is clearly value in incorporating episodic memory into computational models of value-based decision-making (Gershman & Daw, 2017; Lengyel & Dayan, 2007), the leading models are still based on reinforcement learning (RL) principles (Rescorla & Wagner, 1972; Sutton et al., 1998). Instead of recalling episodes of previous rewards, these models assume that rewards are integrated into a scalar value signal that is then used to guide decisions (Sutton et al., 1998; Rescorla & Wagner, 1972; Wilson & Collins, 2019). Developing novel computational models that directly address role of episodic memory in value-based decision-making is critical for understanding experimental data that links sequential decision-making to episodic memory, but also for understanding and ameliorating the decision-making deficits that so often occur in disorders of memory (Gaubert & Chainay, 2021).

There are a variety of computational schemes by which episodic memories can be incorporated into value-based decision-making (Bornstein et al., 2017; Erev et al., 2008; Nicholas et al., 2022; Ramani, 2019). However, these models are either noiseless, while we know that there are fundamental constraints on the fidelity of memory: the brain is not able to perfectly encode, retain, represent, or recall information (Ebitz et al., 2019; Gregory, 1980; Howard & Kahana, 2002; Jurewicz et al., 2022; Lynn et al., 2020; Shourkeshti et al., 2023); or the modeled noise in memory is uniformly random across sampled episodes, and episodes’ outcomes are weighted to produce an analog of RL value (Biele et al., 2009; Erev et al., 2008; Plonsky et al., 2015). While mnemonic noise would certainly hinder some cognitive processes, in the case of decision-making under uncertainty, there is both theoretical (Kirtland et al., 2025; Robbins & Monro, 1951) and empirical (Ebitz et al., 2019; Pisupati et al., 2021) evidence that noise is critical for exploratory discovery and learning. This implies that biologically inspired noise is not only an important consideration in designing and evaluating computational models of episodic decision-making but could potentially be a way to improve model performance.

Here, we build on previous work from our group that introduced minimal episodic decision-making agents that we called “Imperfect Memory Programs” (IMPs) (Ramírez-Ruiz & Ebitz, 2025). We developed IMPs as a thought experiment: a decision-making algorithm that keeps a memory of past rewards to guide choices, without ever calculating value in the RL sense. This prior work proposed a principled answer to a fundamental question: without considering value, which memories should we recall to guide our present decisions? We proposed that the optimal solution should balance error (which increases as the sampled memories are further away in the past) and complexity (which increases as we give more importance to a few specific memories as opposed to uniform importance across memories). The solution to the error-complexity trade-off turned out to be a scheme in which the influence of past rewards on the present choice fell off exponentially. This resembled the pattern commonly seen in biological decision-making (Lau & Glimcher, 2005). Critically, this pattern is generally interpreted as evidence of value-based computations, although the IMPs made their decisions purely through episodic recall, without calculating value. We also reported that this optimal noisy sampling process generated more flexible behavior and slightly better performance than only recalling the last memory perfectly in some classic decision-making tasks.

The major limitation in our prior work on IMPs was that the theoretical optimum of the complexity-error trade-off is only guaranteed by an ever-growing memory of the agent’s interactions with the world. This ultimately means that the algorithm implemented in IMPs is not feasible either in reality (where there should be a price to pay for a large storage of memory) or in simulations (where a maximum window was implemented). Here, we provide a biologically plausible approximation of IMPs. Instead of sampling from a large buffer of reward memories, our novel Lossy Imperfect Memory Programs (LIMPs) keep only one reward memory, but encode this reward only with some probability. In this way, the probability of keeping a specific reward memory falls off exponentially, similar to the original IMPs. This approximation has the added advantage that the probability of encoding a reward can depend on the specific value of the reward, for example, positive rewards can be encoded with higher probability than negative rewards, mirroring asymmetries observed in human reward learning (Adcock et al., 2006; Lefebvre et al., 2017; Palminteri & Lebreton, 2022). These Biased and Lossy Imperfect Memory Programs (BLIMPs) generate flexible behavior that spans the spectrum between matching (take actions proportionally to their probability of reward) and maximizing (take actions that maximize reward). BLIMPs can adapt more quickly than agents built to maximize reward with value computations and perform better than agents that encode past rewards perfectly. These results have implications for understanding the mechanistic basis of sequential decision-making, but also for designing new algorithms for decision-making in uncertain environments.

## Lossy Imperfect Memory Programs (LIMPs)

**Figure 1.**
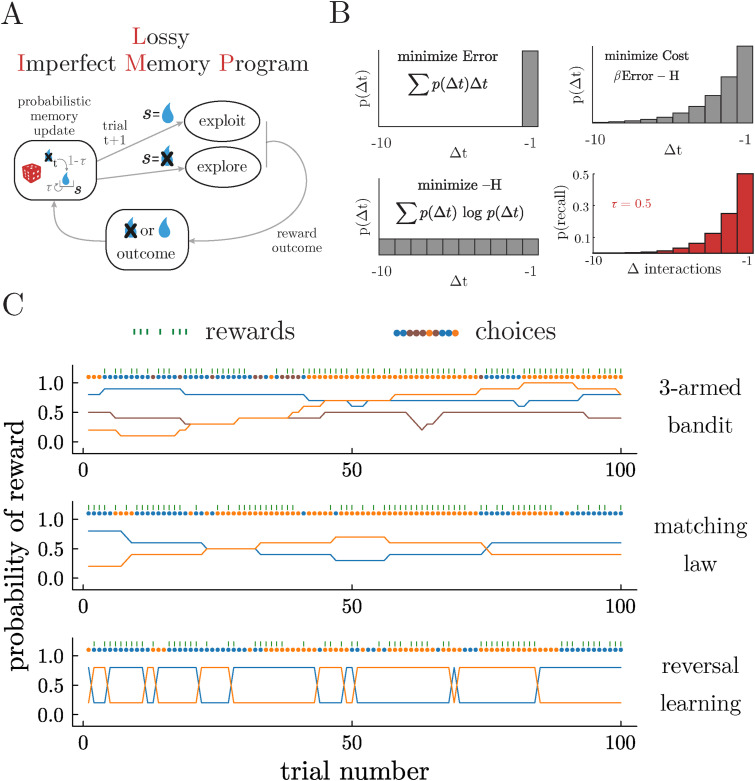
Agents and environments. A) Schematic illustrating the structure of the decision-making process in Lossy Imperfect Memory Programs (LIMPs). B) LIMPs fail to encode reward outcomes in memory with a certain probability, *τ*, in a way that approximates the optimal trade-off between (1) minimizing the average temporal error of the recalled samples (top left) and (2) minimizing the complexity of the sampling process (bottom left). The Boltzman distribution (top right) minimizes the total cost (or free energy) of the sampling distribution, thereby naturally balancing these two objectives. Here *β* becomes the inverse temperature of a process that samples probabilistically from memory. LIMPs approximate this storage process through probabilistically *storing* previous reward outcomes, such that the likelihood of recalling previous rewards approximates the optimal Boltzman distribution (bottom right). C) Examples of the reward schedules from 3 testbeds: a restless 3-armed bandit (top), a matching law task (middle), and a probabilistic reversal learning task (bottom). Delivered rewards and the choices generated by one example LIMP are overlaid

In previous work (Ramírez-Ruiz & Ebitz, 2025), we introduced a class of agents that use memory imperfectly to guide choices: Imperfect Memory Programs (IMPs). Here, we will briefly review the structure and logic behind IMPs in order to lay the groundwork for the novel and biologically plausible extensions that we will develop here (Figure 1A–C).

IMPs make decisions via a two-stage process. First, in the valuation stage, IMPs “remember” a past outcome via a process that samples prior rewards from memory, subject to some noise. Second, in the choice stage, IMPs decide whether to exploit the action or to explore the action space. The noisy memory sampling process is designed to trade-off two objectives (Figure 1B). First, we reasoned that the memory store should maximize the likelihood of recalling relevant past outcomes. In changing environments, the relevance of past information decreases as a function of time, such that recalling outcomes distant in time (Δ*t* with respect to the present), incurs a high error. Second, the recall process should be as minimally complex as possible, maximizing the information that can be sampled from memory. Balancing these two competing objectives by calculating the total cost gives us the Boltzmann distribution (Ramírez-Ruiz & Ebitz, 2025)

(1) $$p\left(R_{\Delta t}\right)=\frac{1}{Z}e^{-\beta \Delta t},$$

where *Z* is the partition function and *β* is the model’s single free parameter, which controls the trade-off between error magnitude and complexity. Although this choice is theoretically motivated (Lynn et al., 2020; McNamara & Houston, 1987), there is also empirical evidence that memory retrieval tends to be exponentially recency-weighted (Barron & Erev, 2003; Biele et al., 2009; Estes, 1976; Howard & Kahana, 2002; Lynn et al., 2020; Ranc et al., 2021), suggesting that the brain may have also struck this balance between error magnitude and complexity.

After drawing a memory according to this sampling rule, IMPs make a deterministic choice based on the sampled outcome. Inspired by recent computational models that suggest that humans make decisions at the level of behavioral policies rather than primitive actions in this task (Harrell et al., 2025; Zid et al., 2025), IMPs use the sampled outcome to choose whether to explore or exploit. If the remembered outcome, *R*_Δ*t*_ was not positive (not rewarded), the agent explores through choosing a new action policy at random

(2) $$p\left({choice}_{t+1}=i|R_{\Delta t}=0\right)=\frac{1}{k},$$

where *k* is the total number of options. If the remembered outcome was positive (rewarded), the agent continues to exploit its current action policy.

(3) $$p\left({choice}_{t+1}=i|R_{\Delta t}=1\right)=\left\{\begin{array}{c} 1,{\text{if choice}}_{t}=i\;\\ 0,otherwise\; \end{array}\right..$$

This decision policy mirrors one that seems to describe human decision-making well (Harrell et al., 2025; Zid et al., 2025), resonates with behavioral evidence that biological decision-makers produce distinct explore and exploit choices in similar tasks, where the exploration resembles random decision-making and exploitation resembles directed, reward-dependent decision-making (Chen, Knep et al., 2021; Ebitz et al., 2018; Laurie et al., 2024). Note that an agent implementing this decision-rule with perfect recall would perform similarly to a win-stay, lose-switch (WSLS) agent (Robbins & Monro, 1951), except that the probability of switching is not equal to 1 when remembering negative reward, but instead depends on the number of arms (*p* (switch) = (*k* − 1)/*k*).

By contrast, we previously reported that the imperfect recall in the IMPs allows these agents some robustness in the face of stochastic rewards and volatile environments (Ramírez-Ruiz & Ebitz, 2025). Further, imperfect recall reproduced an exponential weighting of past rewards on the current choice, a classical result from the animal behavior literature that is typical of value-based decision-making and thought to be indicative of some kind of integration process (Lau & Glimcher, 2005). The major limitation of the IMPs as a class of decision-making models is that the theoretical optimum of the complexity-error trade-off is only guaranteed by an ever-growing memory of the agent’s interactions with the world. This ultimately means that the algorithm implemented in IMPs is not feasible either in reality, where there should be a price to pay for a large storage of memory, nor in our simulations, where a maximum window was implemented.

Therefore, here we develop a biologically feasible approximation to the optimal trade-off implemented in equation (1)—not by imperfectly sampling from a perfect memory, but by introducing biologically realistic encoding errors. We will call this approximation “Lossy IMPs” (LIMPs; Figure 1A–C). Consider an agent that keeps only one outcome in memory, but imperfectly: it has a probability *τ* of failing to encode the latest reward outcome (Figure 1A). This scheme produces a geometric distribution over the probability of retrieving past rewards that falls off with *τ*, such that the probability of a past reward *r* for action *a* surviving in memory for *n*_*a*_ interactions with this action is $\tau^{n_{a}}$ (Figure 1B, red distribution). This scheme is thus, at first glance, a reasonable approximation to the optimal retrieval that we derived in the previous sections and implemented in the IMPs (Figure 1B). Because the geometric distribution is the discrete analog of the exponential, *τ* is inversely related to the IMP’s *β* parameter, controlling the probabilistic decay of previous memories on future choices. In fact, we can compare both parameters from the distribution they induce, $\exp\left(-\beta \Delta t\right)\equiv \tau^{\Delta t}\;\Rightarrow \;\beta \equiv \log\left(1/\tau \right).$

Unlike noisy sampling in IMPs, the noisy encoding of reward in LIMPs does not require a perfect, infinite memory of previous reward outcomes. In fact, given the structure of choice in LIMPs (Figure 1A), they only need to keep one bit of memory: they only sample a new option if the recorded memory for the current option is negative. However, relaxing this assumption does mean that reward outcomes that are not encoded cannot later be retrieved. As a result, we might expect the difference between IMPs and LIMPs to be most apparent for high values of *τ*, given that LIMPs will not be able to encode new values of reward, getting “stuck” in old memories. However, LIMPs also have a degree of flexibility that was not present in the original IMP algorithm. Specifically, introducing imperfections at the encoding stage, rather than the retrieval stage, allows us to account for the possibility of different encoding probabilities depending on the received reward, for example, *τ*_+_ and *τ*_−_ for the case of binary rewards, which we call Biased and Lossy IMPs (BLIMPs). This mirrors experimental evidence that biological decision-makers weigh positive (higher than expected) outcomes more strongly than negative (lower than expected) outcomes (Lefebvre et al., 2017; Palminteri & Lebreton, 2022). These asymmetries are typically modeled as different learning rates for positive and negative outcomes in Rescorla-Wagner-type reinforcement learning algorithms, and here we draw on observations that memory tends to covary with valence and allow positive and negative outcomes to have different probabilities of being stored in memory (Davidow et al., 2016; Rosenbaum et al., 2022; Sharot & Garrett, 2016). Because BLIMPs allow encoding errors to depend on reward outcomes, the noisy encoding scheme investigated here is both an approximation to, and an extension of, the original IMPs algorithm described previously (Ramírez-Ruiz & Ebitz, 2025).

## Testbeds

Following previous work (Ramírez-Ruiz & Ebitz, 2025), we simulated behavior from IMPs, LIMPs and BLIMPs in three sequential value-based decision-making tasks that are common in the neuroscience and psychology literature (Figure 1C). These included a restless bandit task (Chen, Knep et al., 2021; Daw et al., 2006; Ebitz et al., 2018; Pearson et al., 2009), a matching law task (Sugrue et al., 2004), and a probabilistic reversal learning task (Butter, 1969; Chen, Ebitz et al., 2021; Ebitz et al., 2019). Unless otherwise noted, simulations involved 500 sessions (“walks”) of 500 trials each. All agents experienced identical environments.

In each task, choices are made between a set of *k* options, each of which is associated with some probability of reward. Reward probabilities can only be inferred by choosing each option and combining information over multiple samples. The tasks are all uncertain because the reward probabilities are not fixed, but instead evolve over time. This encourages decision-makers to exploit valuable options when they are discovered while also occasionally exploring alternative options that have the potential to become more rewarding at any time.

In the restless bandit task (Figure 1C, top), the reward probabilities of each option *i* are independently updated at each trial *t* according to

(4) $$p\left({reward}_{i,t+1}\right)=p\left({reward}_{i,t}\right)\pm \left\{\begin{array}{cc} step,\; & \text{if }u∼U\left(0,1\right)<hazard\\ 0,\; & otherwise \end{array}\right.$$

where “hazard” is a fixed rate of change ∈ [0, 1], u∼U(0,1) is a draw from a uniform random distribution, and the sign of the step is chosen independently at random for each option on each trial. The hazard rate and step size were both fixed at 0.1, and the number of options was fixed at 3 except as otherwise noted, after (Chen, Ebitz et al., 2021; Ebitz et al., 2019; Laurie et al., 2024; Shourkeshti et al., 2023).

In the matching law task (Figure 1C, middle), reward probabilities are updated according to the same function, but not independently because

(5) $$\overset{k}{\underset{i=1}{\sum }}p\left({reward}_{i,t}\right)≔1$$

for each option *i*. The matching law task is often used to illustrate that biological decision-makers tend to be imperfect reward maximizers: more likely to allocate their choices in proportion to the rate of reward than to choose the best option. Matching law tasks are typically 2-alternative; we followed that convention here.

The probabilistic reversal learning task (Figure 1C, bottom) is another common 2-alternative task in decision-making experiments in rodents, with the specific aim of analyzing the mechanisms that permit them to adapt to sudden changes in the environment. As in the matching law task, reward probabilities are symmetrical such that one option is high value and the other is low value. However, here the high and low values are fixed, often at *p* (reward|high) = 80% and *p* (reward|low) 20%, with the identity of the high and low values swapping at specific reversal points.

## Results

### LIMPs Replicate Key Signatures of IMPs

A typical class of sequential decision-making algorithms updates the value of choosing an action *Q*(*a*) with a delta rule, where after receiving a reward *R*_*t*_ at time *t*, the value is updated at time *t* + 1,

(6) $$Q_{t+1}\left(a\right)=\left(1-\alpha \right)Q_{t}\left(a\right)+\alpha R_{t},$$

which makes explicit that action values *Q*(*a*) are the *α*-weighted average of value at the previous time step and the newest reward. This implies that the weight of past rewards falls off exponentially in these models, mirroring the pattern that is commonly seen in biological decision-makers (Lau & Glimcher, 2005).

Previous work showed that IMPs generate value-like reward history kernels without ever calculating value, because of their memory errors. To determine whether the same was true in LIMPs, we measured the LIMPs’ reward history kernels via simulating LIMPs in a 2-armed bandit task and fitting a logistic regression model,

(7) $$log\frac{p\left(c_{t}=1\right)}{p\left(c_{t}=-1\right)}=\beta_{0}+\overset{N}{\underset{i=1}{\sum }}\alpha_{i}c_{t-i}+\overset{N}{\underset{i=1}{\sum }}\phi_{i}c_{t-i}r_{t-i}+\eta ,$$

where *c*_*t*−*i*_ is 1 if the first option is chosen on trial *t* − *i* (−1 if the second is chosen), and *r*_*t*−*i*_ is 1 if they were rewarded on that trial (0 otherwise). Together, the *ϕ*_1:*N*_ parameters represent the unique effect of previous rewards on the log odds of choice, beyond the contribution of choice history (*α*_1:*N*_) and bias (*β*_0_). Models were fit via ridge-regularized maximum likelihood (*λ* = 1). To determine if the influence of previous rewards decayed exponentially quickly, we fit a 3-parameter exponential curve, *Ae*^−*Bx*^ − *C*, to *ϕ*_1:*N*_. Here, *A* represents a scaling parameter, *B* is the decay rate of the influence of previous rewards, and *C* is an offset.

**Figure 2.**
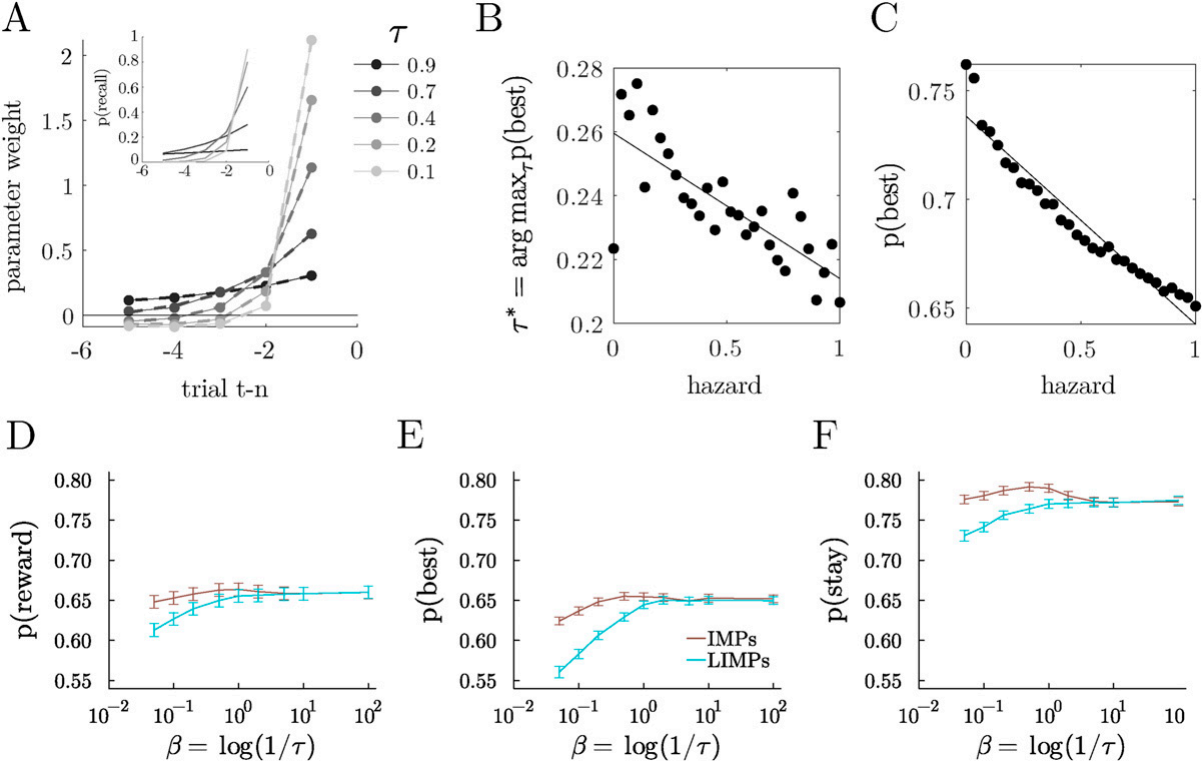
LIMPs integrate reward history via imperfect memory. (A) Reward history kernels for the example LIMPs, with exponential fits overlaid (inset). Geometric (discrete exponential) distributions illustrating the probability of holding a previous reward in memory as a function of the number of interactions with this arm for 5 example LIMPs with different *τ*. (B) The *τ* that maximizes the probability of choosing the best option, plotted as a function of volatility (hazard), identified via grid search (20 log-distributed bins ∈ [10^−2^, 1]). (C) Probability of choosing the best option for the optimal LIMP, plotted as a function of volatility. (D-F) Probability of obtaining reward (D), choosing the best option (E) and staying with the same option (F) for IMPs and their approximation with LIMPs, as a function of their associated noise parameter

Like the IMPs they were designed to approximate (Ramírez-Ruiz & Ebitz, 2025), LIMPs reliably generated reward history kernels that were well-described by exponential decay (Figure 2A; median *R*^2^ = 0.99 across 200 random simulated LIMPs, *τ* ∼ *U* (10^−2^, 1)). The decay in the reward history kernel also changed systematically as a function of *τ* (Figure 2A). Although exponentially decaying reward history kernels are often taken as evidence of RL-like value computations, LIMPs generated similar kernels because of the probabilistic memory encoding process, rather than any value calculations.

If LIMPs’ imperfect memory accomplishes something like reward integration, then in less volatile environments, where longer reward history integration offers an advantage, the optimal *τ* should increase, indicating a longer “integration” period. Similarly, when volatility is high, a shorter “integration window” would allow for more flexibility and adaptation. To test these predictions, we simulated LIMPs in the restless bandit with varying hazard rates. We found that the optimal *τ* scaled with volatility (Figure 2B). This observation suggests that the imperfect memory process in LIMPs functioned like the reward history integration in delta-rule learning agents. Further, we found that when volatility is low, LIMPs that used longer “integration” windows were able to exploit the stability of the environment and perform better (Figure 2C). Thus, the mnemonic noise in LIMPs, like in IMPs, was able to accomplish something like reward integration.

Despite this parallel, LIMPs are not a perfect approximation of IMPs, so we also sought to understand when LIMPs are a good approximation to IMPs from the perspective of task performance. To determine when these algorithms diverged, we simulated IMPs and LIMPs in the restless 3-armed bandit, with the usual hazard rate of 0.1 as defined in section “Testbeds”. We found that IMPs and LIMPs differ mostly at high values of *τ* (low values of *β*). This appeared to occur because LIMPs with high values of *τ* are only rarely able to encode new values of reward and thus get “stuck” in old memories. Nonetheless, the mapping between parameters *β* ≡ log (1/*τ*) showcases how the distributions of both agents are related, and shows that the approximation is good for high values of *β*, corresponding to high values of the successful encoding probabilities 1 − *τ* (Figure 2D–F).

### BLIMPs Perform Better Than IMPs, LIMPs, and Perfect Encoding Agents

Our previous work showed that IMPs are a sufficient and simple strategy to perform classic decision-making tasks without ever calculating value (Ramírez-Ruiz & Ebitz, 2025). Furthermore, the imperfect recall of IMPs provided a better flexibility and reward maximizing behavior than perfect recall agents like Win-Stay Lose-Shift (WSLS) agents (Ramírez-Ruiz & Ebitz, 2025). In this way, IMPs provided an interesting generalization of WSLS agents, though not a big improvement in performance. LIMPs, as an approximation to IMPs, were able to achieve similar levels of performance at some parameter values, but never outperformed IMPs (Figure 2D–F). We therefore next asked if there was a way to improve the performance and flexibility of LIMPs via adding a bias known to exist in human decision-making: namely, asymmetrical effects of positive and negative or omitted rewards (Lefebvre et al., 2017; Palminteri & Lebreton, 2022). We reasoned that this kind of asymmetry might make LIMPs better able to adapt to noisy environments via making them selectively less sensitive to certain kinds of noisy reward feedback.

**Figure 3.**
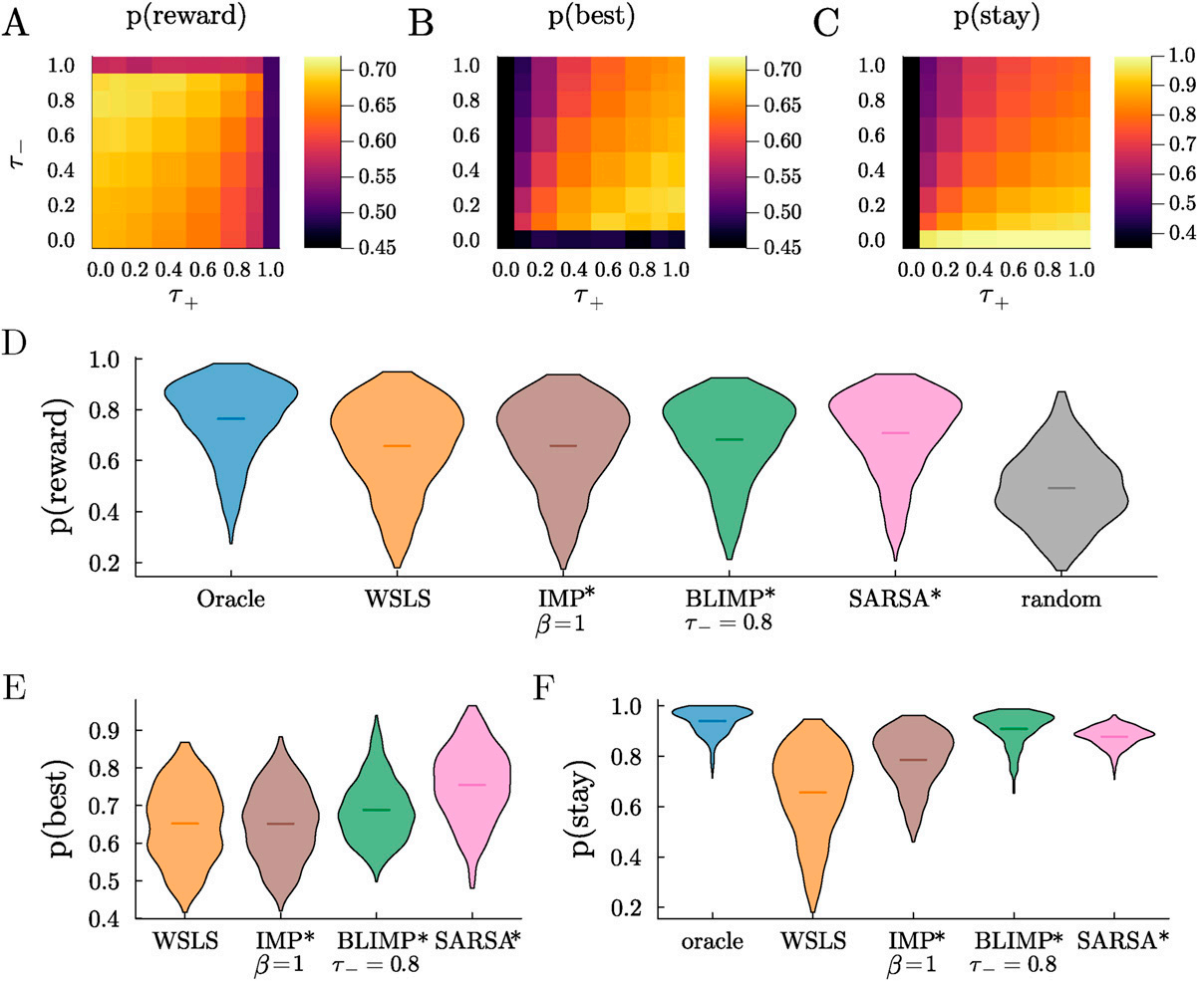
LIMPs and BLIMPs perform a restless multi-armed bandit task without perfect memory. Probability of (A) obtaining reward, (B) choosing the best arm, and (C) sticking with the same arm across sessions, as a function of the probability of failure to encode positive (*τ*_+_) and negative (*τ*_−_) rewards (LIMPs correspond to diagonal *τ*_+_ = *τ*_−_). Note that the colorbar for stay has a different scale for better visualization. (D) Probability of obtaining reward for various agents, with their free parameters optimized for this task. (E) Same as D, for the probability of choosing the objectively best option (or any of the best options). Oracle (always 1) and random ( ≈ 1/k) are not shown. (F) Same as D for the probability of stay. Random (always 1/k) is not shown

Asymmetrical reward effects is most commonly modeled as asymmetries in learning rates in RL models, but, in LIMPs, the natural way to weigh rewards differently was to make the encoding probability of reward depend on outcome. We called this variation Biased LIMPs (BLIMPs). In contrast to LIMPs’ single encoding parameter, *τ*, BLIMPs have two independent free parameters: *τ*_+_ and *τ*_−_, the probability of failing to encode positive rewards and negative (omitted) rewards respectively. We discovered that the BLIMPs that best perform the 3-armed bandit task, in terms of choosing the best option and obtaining reward, encode positive rewards perfectly $\tau_{+}^{*}=0$ , but encode negative rewards quite imperfectly $\tau_{-}^{*}=0.9$ (Figure 3A and B).

The BLIMPs consistently outperformed other agents. For example, they obtained more reward and were more likely to choose the best option than LIMPs (i.e., in Figure 3A and B, off-diagonal agents where *τ*_+_ ≠ *τ*_−_ perform better than on-diagonal agents, where *τ*_+_ = *τ*_−_, corresponding to LIMPs). The optimal BLIMPs also obtain higher average reward and choose the best option more than the best IMPs, despite the fact that the latter were lossless and unbiased (Figure 3D, p (Reward BLIMPs) > p (Reward IMPs) in more than 83% of walks, and Figure 3E, p (Best BLIMPs) > p (Best IMPs) in more than 75% of walks). BLIMPs also tended to persist more in choosing the same option in all walks than these other agents (Figure 3F, p (stay BLIMPs) > p (stay IMPs) = 1.0). This is an important observation given that switching has both time and energetic costs (Shourkeshti et al., 2023; Wylie & Allport, 2000). These results imply that a biased and lossy memory-based decision-making algorithm would not only achieve higher rates of performance than perfect recall and also do so more efficiently.

To benchmark the BLIMPs’ performance against non-memory-based algorithms, we compared them with a variety of reference strategies. These included a Win-Stay Lose-Shift (WSLS) strategy, a simple heuristic often used as a benchmark in diverse fields where agents switch away from a previously rewarded option when not rewarded (Robbins & Monro, 1951). We also simulated an oracle (that knows the probability of each arm and always selects the best), a random agent (which chooses an arm uniformly randomly), and a reinforcement learning algorithm (SARSA (Sutton et al., 1998)). SARSA updates the value *Q*(*a*) of an arm *a* after receiving reward *R* at time *t*, and sampling another arm *a*′ from its policy

(8) $$Q_{t+1}\left(a\right)=Q_{t}\left(a\right)+\alpha \left(R_{t}+\gamma Q_{t}\left(a^{\prime }\right)-Q_{t}\left(a\right)\right),$$

where *α* is a learning rate and *γ* is a discount factor. Then, the SARSA agent defines a probability *π*(*a*) of choosing an arm *a* using the action value *Q*(*a*), as a softmax distribution

(9) $$\pi \left(a\right)=\frac{1}{Z}\exp\left(\beta_{\text{SARSA}}Q\left(a\right)\right),$$

where *β*_SARSA_ is an inverse temperature parameter that controls the noise for the SARSA agent, and *Z* is the normalizing partition function. Given the non-stationarity nature of the tasks, this parameter allows SARSA agents to keep exploring the options, which helps them discover changes in the environment.

The optimal BLIMPs performed better than WSLS agents in the restless 3-arm bandit task, in both probability of obtaining reward and choosing the best arm (p (IMP > WSLS) >0.73 for both metrics). Both WSLS and BLIMP agents underperformed SARSA (p (SARSA > BLIMP or WSLS) > 0.85 for both metrics), with optimized parameters *α* = 0.8, *γ* = 0.9, and fixed *β*_SARSA_ = 10 (Figure 3D). All agents perform better than chance (*p* < .001). For the parameters of this task, both the oracle and the optimized SARSA agent tended to persist in the same option, making repeated stay decisions (Figure 3D, probability of staying is higher for these agents than for either WSLS or the IMPs). Similarly, the BLIMPs stay more than WSLS agents in all the walks tested (p (BLIMP stay > WSLS stay) = 1.0).

### BLIMPs Cover the Matching and Maximizing Spectrum

**Figure 4.**
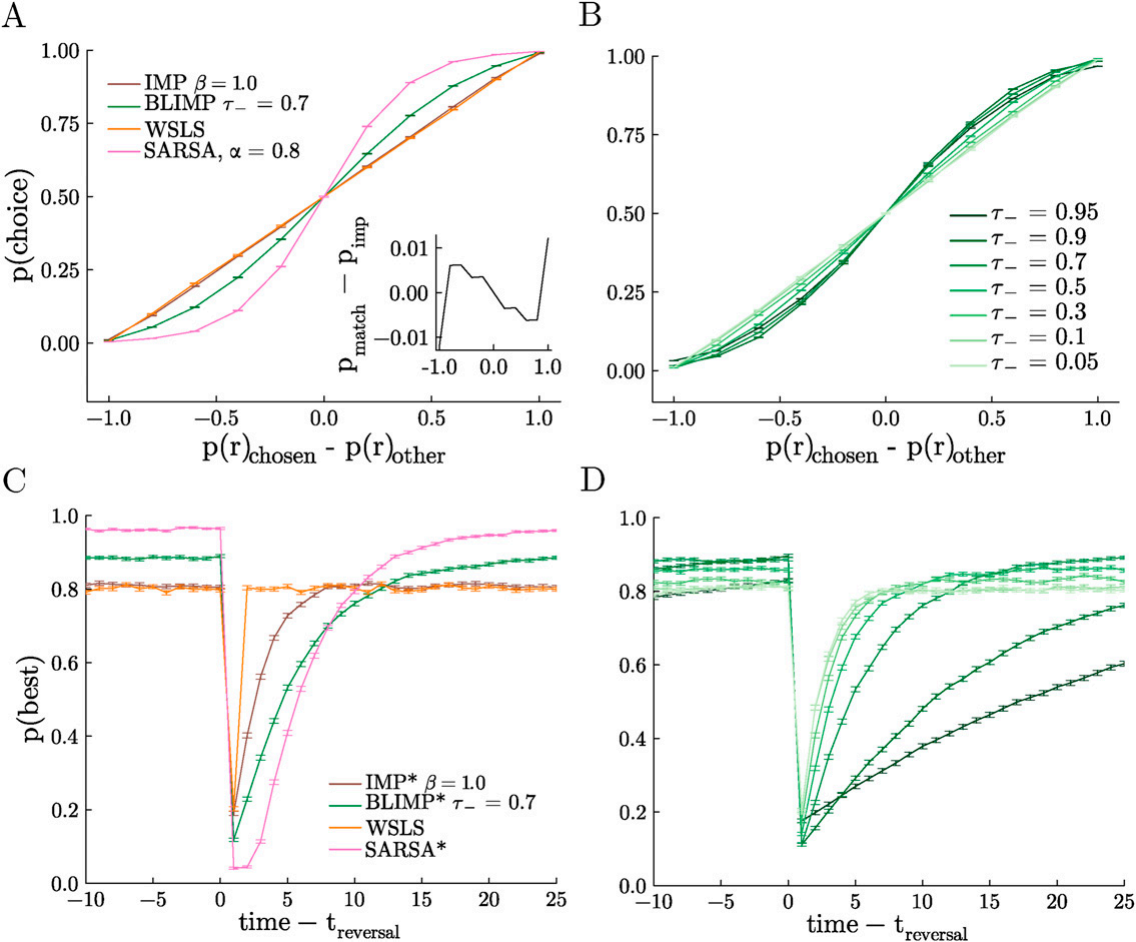
BLIMPs adapt flexibly between matching and maximizing. (A) Probability of choosing a particular arm as a function of that arm’s probability of reward minus the probability of reward of the unchosen arm (matching task). Inset: probability of choice of a perfect matching strategy (a straight line) minus the probability of choice for the IMP agent as a function of the arm’s probability of reward minus the probability of reward of the unchosen arm. (B) Same as A, for many values of *τ*_−_ and *τ*_+_ = 0 for BLIMPs. (C) Probability of choosing the best arm as a function of time relative to the onset of a reversal event (reversal task). (D) Same as C, for many values of *τ*_−_ and *τ*_+_ = 0 for BLIMPs. Legend same as in B. Tasks described in Section 3, with a hazard rate of 0.02. All agents received the same random walks in each session

Under some circumstances, biological decision-makers tend to match the relative rate of reward of their options rather than maximize their reward by consistently choosing the best option (Fantino, 1981; Sakai & Fukai, 2008; Soltani & Wang, 2006; Sugrue et al., 2004). In previous work, IMPs were tested in a matching law testbed to determine the degree to which they matched versus maximized reward. We found that IMPs tended to match: they allocated their choices in proportion to the rate of reward associated with each option. However, whereas the WSLS agent is a perfect matcher by design, there was a very slight tendency towards maximizing in IMPs when compared to this reference (Figure 4A, inset). Given the improved performance of BLIMPs in the restless 3-armed bandit, we therefore next asked if the biases present in the BLIMPs made them more likely to maximize than IMPs (and therefore LIMPs). Indeed, BLIMPs that encode positive rewards perfectly (*τ*_+_ = 0), yet negative rewards quite imperfectly (*τ*_−_ > 0), show a qualitatively higher degree of maximizing (Figure 4A and B). The degree of maximizing was proportional to *τ*_−_ such that sweeping the probability of encoding negative rewards gave a spectrum between matching and maximizing behavior (Figure 4B). Note that perfect encoding here (*τ*_+_ = *τ*_−_ = 0) corresponds to a perfectly matching, Win-Stay Lose-Explore (WSLE) strategy and imperfect encoding improves performance towards a maximizing agent. In order to compare BLIMPs against a true value-integrating agent, we also simulated matching law behavior from an optimized SARSA agent (*α* = 0.8, *γ* = 0.9), and found a stronger maximizing effect in SARSA than in any parameterization of the BLIMPs. Thus, although BLIMPs maximize much better than IMPs or LIMPs, they did not maximize to the full extent possible in this task.

### BLIMPs Trade-Off Flexibility and Stability

In biological brains, there is a natural trade-off between the ability to persist in stable environments and the ability to adapt to change (Ebitz et al., 2019; Liljenström, 2003). IMPs can resolve this trade-off, which was shown in a probabilistic reversal learning task in which stable periods (where one option is clearly more valuable than the other) are interspersed with “reversals” (where the values flip). However, the performance of IMPs was fairly similar to WSLS agents: they are able to perform marginally better during stable periods, but adapt more slowly at change points (Ramírez-Ruiz & Ebitz, 2025). Given that BLIMPs show better performance in both the 3-armed restless bandit and the matching task, we therefore next tested them in this reversal learning task to see how biased reward encoding affects the ability to adapt to reversal events.

During stable periods, some BLIMPs were able to outperform WSLS agents and IMPs (Figure 4C). This was because they were better able to persist in choosing the high value option despite the noisy reward, given by their probabilistic encoding of negative rewards, but perfect encoding of positive rewards. By contrast, at reversals, these same BLIMPs learned less quickly than the WSLS agents and the IMPs. To estimate what a value-integrating agent would do, we again simulated behavior from the optimal SARSA agent (*α* = 0.8, *γ* = 0.4). Although the BLIMPs were less capable than SARSA during the stable periods, they adapted faster at reversals—already beginning to reverse after the first omitted reward, whereas SARSA required several omitted rewards in order to begin adaptation. In addition, BLIMPs can again show flexible behavior depending on their probability of encoding negative rewards, from matching WSLE when negatives are encoded perfectly *τ*_−_ = 0, to slower adaptation but better performance at higher levels of *τ*_− _(Figure 4D). This relationship was non-monotonic, with an optimal *τ*_−_∼ 0.7. In sum, BLIMPs were again able to solve a classic sequential decision-making task and their performance levels were in between the extremes of a memory-less agent with perfect access to previous reward and a full reinforcement learning agent, with the bonus of being able to exhibit behavior close to those extremes by tuning their free parameters.

## Discussion

The ability to robustly store and retrieve information about past interactions with the world is crucial for adaptive behavior and recent research has increasingly implicated memory processes in reward-based decision-making (Bornstein et al., 2017; Cohen et al., 2022; Gershman & Daw, 2017; Nicholas et al., 2022; Plonsky et al., 2015; Ramani, 2019). Because memory is imperfect, previous work explored a simple, yet surprisingly competent model for decision-making that incorporates a “faulty” memory. Imperfect Memory Programs (IMPs) have a memory system that provably trades off the cost of retrieval errors and the cost of high complexity. While IMPs showed characteristics that are more aligned to certain natural and normative decision-makers and do well in classic decision-making tasks under uncertainty, their performance and flexibility was not very dissimilar to agents with perfect memory, and their implementation required a large and ever-expanding memory store (Ramírez-Ruiz & Ebitz, 2025).

In this paper, we made an approximation of this optimal trade-off that probabilistically stores the last interaction with the world. Despite having only a one-bit memory, these Lossy IMPs approximated the original IMPs and did so especially when encoding probabilities were high. This approximation allowed us to develop a specific and biologically inspired variation, where rewards are encoded with different probability depending on their value. We showed that asymmetrical encoding of rewards and reward omissions leads to higher performance than IMPs in all the tasks explored in the original work (Ramírez-Ruiz & Ebitz, 2025), faster adaptability and better maximizing than symmetrical encoding probabilities. This work shows that value computations are not necessary to replicate the exponential weighing of rewards of biological decision-makers, and that a simple, lossy and biased memory process is sufficient to generate a wide spectrum of flexible decision-making behavior.

A memory retrieval system that trades off error and complexity costs also improves performance in structure learning (Lynn et al., 2020). That work showed how imperfect memory can enhance generalization because it permits smoothing in learned associations in time, and how humans exhibit these types of “errors” that allow them to generalize over hierarchically structured networks. IMPs demonstrate that the same errors applied to reward learning help agents perform a rudimentary form of value integration. Given that human behavior is consistent with hierarchical reinforcement learning (Eckstein & Collins, 2020), imperfect memory may be a good candidate mechanism for smoothing reward associations in such hierarchically organized spaces.

Recent work has explored the idea of endowing reinforcement learning models with episodic or working memory to improve learning in high dimensional spaces, alleviate resource constraints, and to explain human choice behavior more satisfactorily (Biele et al., 2009; Bornstein et al., 2017; Collins & Frank, 2012; Erev et al., 2008; Gershman & Daw, 2017; Mattar & Daw, 2018; Patel et al., 2020). These previous works focused on using memory to aid RL algorithms in calculating value more accurately in order to improve their generalization and performance. In contrast, our approach poses a different kind of thought experiment, asking what happens when we do not start with the assumption that value is calculated at all. IMPs demonstrate that noisy samples from episodic memory can be sufficient to imply that value integration is occurring even when it is not (Ramírez-Ruiz & Ebitz, 2025) and here we found that the noisy encoding process implemented in LIMPs is also sufficient to generate the exponential reward history kernels thought to be diagnostic of value computations. While humans and other animals may indeed use RL-like algorithms to solve sequential decision-making problems, our results show that even minimal episodic recall algorithms can achieve remarkable results when we imbue them with principled forms of noise.

In most cognitive models, noise is generally only added as an error term (with limited but important exceptions (Findling et al., 2021; Findling & Wyart, 2021; Lynn et al., 2020)). However, it is also possible that the noise in real biological systems serves an important function. Indeed, stochastic memory has recently been shown to be more powerful than deterministic memories in partially observable environments (Kirtland et al., 2025). These and our results suggest that there may be practical value in exploiting stochastic memories, showing how memory errors can actually improve performance in partially observable, changing environments. Developing cognitive models that meaningfully incorporate this kind of noise could be the key to determining when the constraints on the brain are truly a limitation and when they serve a computational purpose.

Another contribution of this work was to show that a system that encodes reward outcomes both probabilistically and asymmetrically improves performance, compared to a symmetrical and perfect memory system. This is an important feature because there is now considerable evidence that biological decision-makers weigh positive (higher than expected) and negative (lower than expected) outcomes differently, with positive rewards typically overweighted with respect to negative rewards (Lefebvre et al., 2017; Palminteri & Lebreton, 2022). Although these asymmetries are typically modeled as different learning rates for positive and negative outcomes in delta-rule reinforcement learning algorithms (Daw et al., 2002; Frank et al., 2009; Frank et al., 2007; Niv et al., 2012), here we assume that positive and negative outcomes have different probabilities of being stored in memory, consistent with numerous observations in the cognitive science literature (Adcock et al., 2006; Biele et al., 2009; Davidow et al., 2016; Katzman & Hartley, 2020; Rosenbaum et al., 2022; Sharot & Garrett, 2016). In the context of RL algorithms, theoretical work has previously suggested that asymmetrical learning rates can improve performance (Cazé & van der Meer, 2013). This work compliments this previous work by (1) extending this result into environments that are typical of the psychology and neuroscience literature and (2) demonstrating that this flexibility can be achieved when asymmetry is incorporated into an episodic learning model via shaping the probability of reward encoding.

Ultimately, IMPs, LIMPs, and BLIMPs are simplistic, intended primarily as thought experiments about the potential effects of mnemonic noise on episodic decision-making. These models are not meant to be viewed as either high performance algorithms or viable alternatives to the leading cognitive models. Nevertheless, we believe that additional work on this agent class could have implications for artificial intelligence and cognitive modeling research. First, the stochastic retrieval and encoding of episodic memories could be extended to actions and states and not just rewards. Agents with stochastic retrieval in all 3 domains might be able to generalize better in complex and hierarchically structured environments (Lynn et al., 2020). Second, our work focused on standard decision-making tasks in the literature that deal with binary outcomes. Extending this work to settings with continuous-valued reward and higher dimensional problems would be beneficial to understand when stochastic and asymmetrical memories are adaptive and when they are costly. Given that biological brains have errors in both memory encoding and recall, determining how and when these errors influence performance is a promising avenue of research.
